# Mass Spectrometry–Based Proteomics for the Masses: Peptide and Protein Identification in the Hunt Laboratory During the 2000’s

**DOI:** 10.1016/j.mcpro.2024.100866

**Published:** 2024-10-22

**Authors:** Jessica R. Chapman

**Affiliations:** Department of Pathology and Laboratory Medicine, Memorial Sloan Kettering Cancer Center, New York, New York, USA

**Keywords:** electron transfer dissociation, MHC peptides, histones, O-GlcNAc, phosphorylation, ion-ion reactions

## Abstract

There has been a rapid increase in the number of individuals utilizing mass spectrometry–based proteomics to study complex biological systems and questions since the start of the 2000’s. Building off the advancements in ionization and liquid chromatography scientists continued to push towards technology that would enable in-depth analysis of biological specimen. Donald F Hunt and the Hunt laboratory were major contributors to this effort with their work on improving upon existing Fourier Transform MS, development of electron transfer dissociation, and continued work on ion-ion reactions to improve intact protein analysis. Collaboration with other instrumentation laboratories and instrument companies led to the sharing of technology and eventual commercialization providing greater access. Additionally, the Hunt laboratory spread the gospel of MS-based proteomics through collaborations that lasted decades with other scientists who were experts in immunology, cellular signaling, epigenetics, and other fascinating fields. This article attempts to highlight the many contributions of Don and the Hunt laboratory to peptide and protein identification since the year 2000.

It is my honor to highlight some of the contributions of Donald F Hunt and the Hunt Laboratory to peptide and protein identification *via* mass spectrometry (MS)-based proteomics from approximately 2000 to present. These years were packed full of developments in this field and Don’s contributions are not minor. The overall trend during these years was the rapid expansion of MS-based proteomics into biological and medical research.

One major driving force was the commercialization and therefore greater accessibility of robust instrumentation with diverse ionization, fragmentation, acquisition, and detector options making advanced methodology available to the masses. Additionally, development and optimization of pre-analytical processing, including sample preparation methods, improved the outcomes of MS-based proteomics experiments. This made it possible to use these techniques for the study of complex biological questions. Lastly an effort to build a community in which data and expertise is shared *via* consortia and collaborations allowed for faster dissemination and implementation of knowledge. Of course, none of these things could be possible without the enthusiastic contributions of the many mass spectrometrists who have built this field and those who continue this effort. Don, with Jeffrey Shabanowitz as his copilot, built a legacy that I and other alums strive to continue in our own careers.

In 2002, Don provided his personal insights on the state of proteomics in this commentary (1). He discussed the development of methodology for the characterization of proteins in complex mixtures, phosphoprotein/peptide identification, and the analysis of differentially expressed proteins in healthy and disease cells as his broad areas of research concentration. These areas of interest are what drove the pre-analytical method and instrumentation advancements in the laboratory leading to technology that benefited the greater proteomics community. Additionally in that piece, Don shared that he saw the future of proteomics leading to major changes in the way we diagnose, treat, and prevent disease and noted the possibility of analyzing diverse biospecimens using MS technology. If interested, read the article in this edition titled “Validation of a mass spectrometry-based proteomics molecular pathology assay.”

This overview is deeply personal as this timeline encapsulates my tenure (2007–2011) under Don and Jeff's mentorship and my career as a mass spectrometrist thus far. Below is intended to serve as a highlight reel of the Hunt Laboratory over the past 24 years. Many of my colleagues will provide more in-depth discussions and reviews of specific areas of Hunt laboratory contributions to the advancements in peptide and protein identification with MS.

## Instrument Development

Analysis of biomolecules by MS had become much easier as we approached the new millennium due to major strides taken in the previous decades including the development of electrospray ionization (ESI) and matrix-assisted laser desorption ionization as these soft ionization methods provided a means to ionize more labile analytes (2, 3, 4). Additionally, advances in liquid chromatography (LC) specifically scaling down to micro and nanoflow levels allowed for coupling of LC to ESI and therefore real-time separation of mixtures (5).

Mass spectrometers used in biological research in the late 1990’s were largely either able to provide accurate mass information in the case of time-of-flight or high-quality fragmentation spectra with lower mass accuracy such as on triple quads and quadrupole ion traps. This often meant a sample was analyzed on multiple instruments to obtain a more complete data set or an incomplete analysis was performed due to limited data. Fourier transform ion cyclotron resonance MS (FTICR MS) was a powerful tool that could be utilized for high mass accuracy, high mass resolution MS1, and MS/MS spectra. The development of external ion injection in the 1980’s with the addition of the quadrupole (QFTMS) allowed for interfacing with ESI (6, 7, 8) and in the 1990’s (9), the implementation of external ion accumulation made it possible to integrate FTICR MS with LC.

In 2000, the Hunt lab published a paper demonstrating the abilities of an in-house built QFTMS configured with an ESI source and external ion accumulation in an octupole ion trap (10). The QFTMS was interfaced with microcapillary LC using columns with integrated nano spray emitters and a split flow LC set up. This instrument combined the technologies developed over the past decades by others in the field and incorporated a “peak parking” technique utilizing a remote-controlled switching valve to move between high and low flow modes. This technique, which had previously been used in the lab (11), allowed the flow to be lowered as peptides eluted, widening the chromatographic peaks, improving ionization efficiency, and allowing for collection of IRMPD MS/MS spectra at subfemtomole levels with high mass resolution (>5000) and mass accuracy of less than 10 ppm. This publication demonstrated the ability to perform high-resolution chromatographic time-scale analysis of proteins and peptides from complex mixtures.

**Fig. 1:**
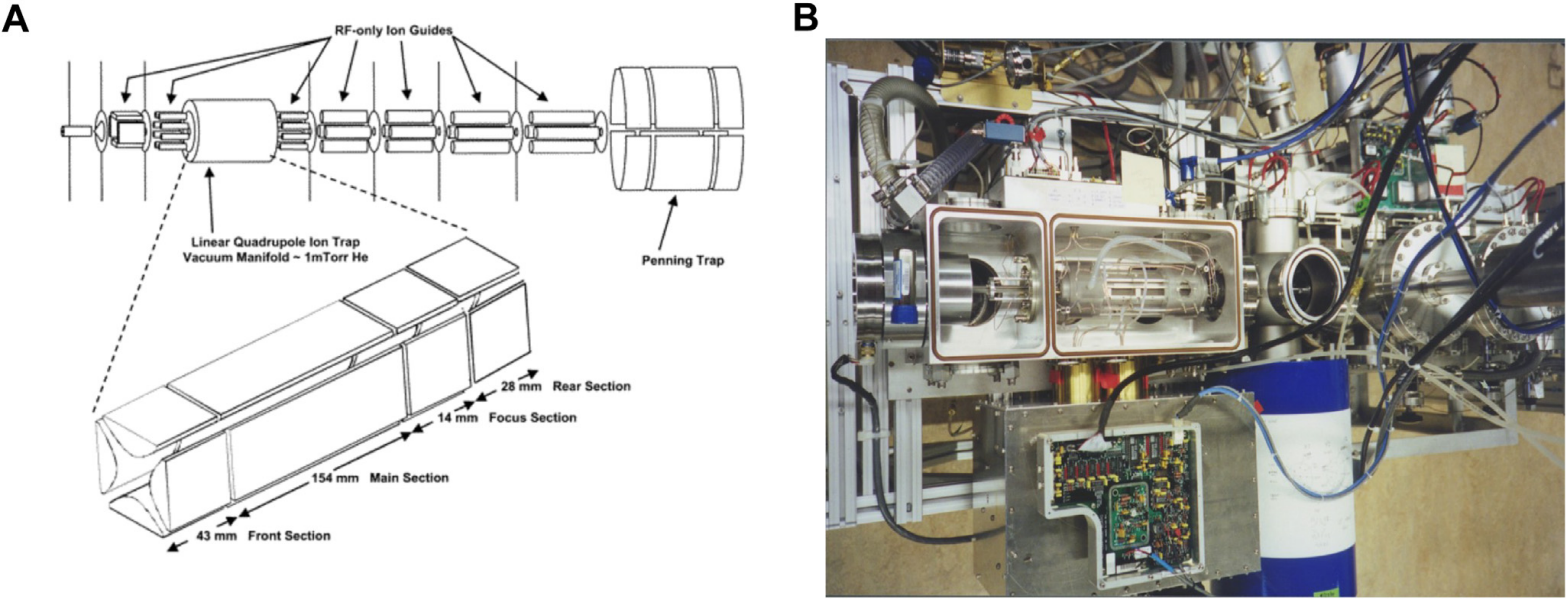
**Prototype linear quadrupole ion trap/Fourier transform mass spectrometer, QLT-FTMS.***A*, instrument schematic reused with permission from ACS Publications. *B*, photograph of the QLT-FTMS from above.

Not long after that publication, a QFTMS instrument built in the laboratory in which the octupole was replaced by a linear quadrupole ion trap (QLT) for ion accumulation (12) (Fig. 1). Specifically, the ion trap used was the same as in the commercial linear ion trap (LTQ) instrument released in 2002. The linear trap was chosen over a 3-D quadrupole trap due to the greater ion capacity. It also increased the scanning speed making this a true chromatography timescale instrument (1 scan/sec) and not requiring the “peak parking” LC technique as previously utilized. Additionally, this instrument surpassed previous instruments with mass accuracy of 1 to 2 ppm and detection at low attomole levels. This instrument was built in 2002 (published in 2004) as a proof of concept for the commercial instrument, the Finnigan LTQ-FT. The LTQ-FT was released by Thermo Scientific in Fall 2003, highlighting the on-going collaborative relationship started many years before between Finnigan and the Hunt Laboratory as discussed by colleagues elsewhere in this volume (Story *et al*). A Finnigan LTQ-FT was installed in the Hunt laboratory in 2003 and was quickly put to work (12).

The contributions of Don and others in the field accelerated the timeline in which these high-resolution instruments were more widely accessible. The commercial instrument provided mass accuracy, MS/MS with automated acquisition, and LC interfacing which allowed for peptide and therefore protein identification in complex biological specimens.

## Electron Transfer Dissociation and Beyond

In the early 2000’s, collision activated dissociation (CAD) had become the primary fragmentation method for peptide characterization by MS and worked well for smaller, lower charged peptides that are commonly produced *via* standard “bottom up” workflows utilizing the endoproteinase trypsin. However, trypsin does not always provide sufficient sequence coverage for proteins of interest, and other endoproteinases can produce longer peptides with multiple basic residues and higher charge states (greater than z = 3), which do not provide sufficient fragment ion coverage *via* CAD. In addition, CAD of peptides with phosphorylation and other labile post translational modifications (PTMs) leads to the loss of the modification and minimal fragmentation along the peptide backbone. Although the signature loss of phosphoric acid is diagnostic, the lack of sequence information limits confident peptide identification.

In contrast, electron capture dissociation (ECD) is able to preserve labile PTMs, fragment along the peptide backbone in a sequence-independent manner, and occurs on a millisecond time scale (13). However, ECD was restricted to use with FTICR MS due to technical challenges of implementation on other MS instrumentation (14). Don initially wanted to implement ECD on the commercial LTQ-FT, but Jeff convinced him it was not worth the effort. First, it would be challenging to add the electron source and secondly the available electron sources were not very bright. It would take 1 to 2 min to accumulate sufficient electrons for the dissociation reaction. Discussions continued and at some point, Jarrod Marto suggested trapping anions and performing electron transfer. Don was excited by the potential to develop a fragmentation technology that had the benefits of ECD but could be implemented on more widely accessible instrumentation including QLT or QTOF and the hybrid FTMS instrumentation (LTQ-FTMS).

**Fig. 2:**
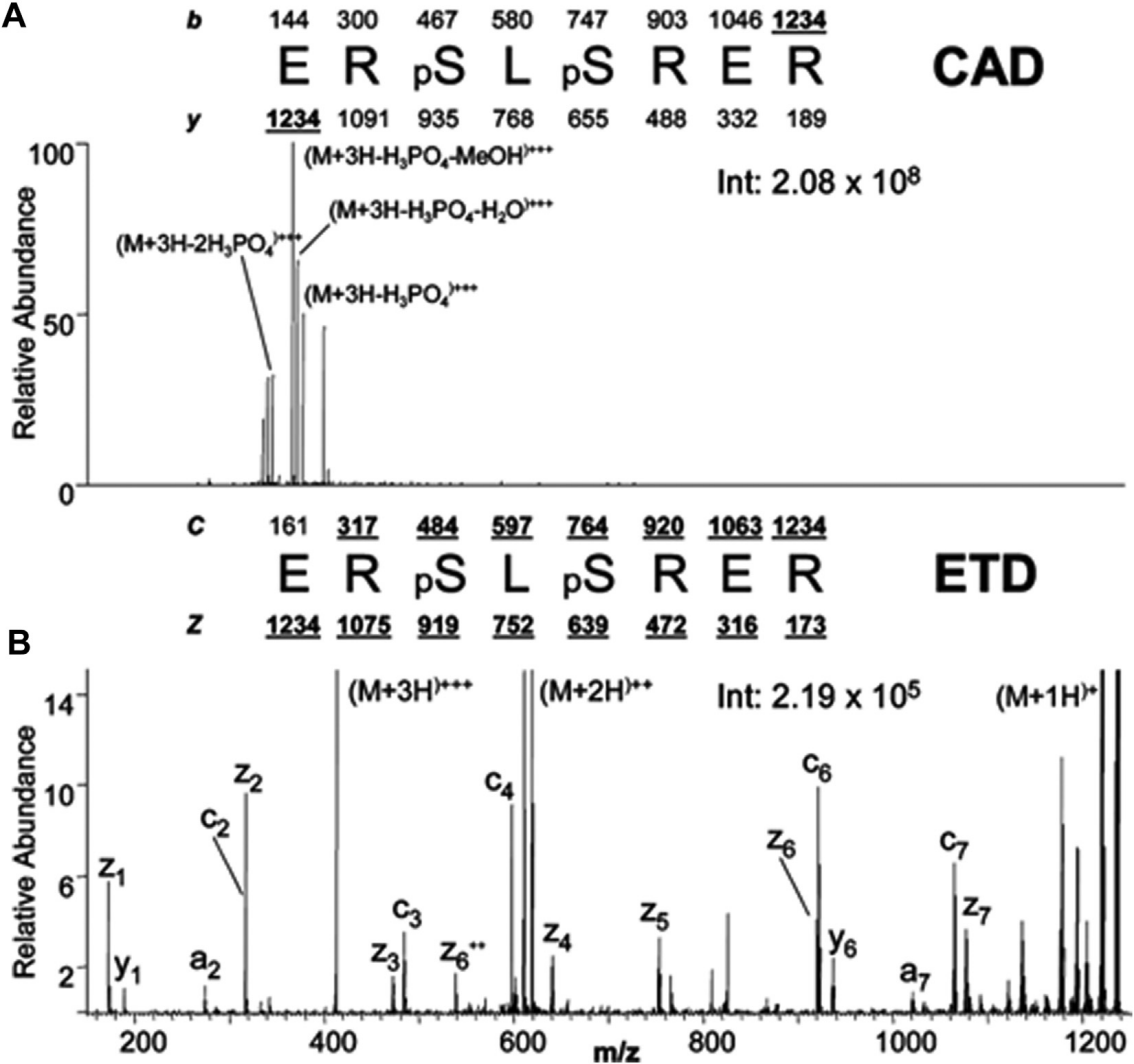
**Comparison of single-scan (500- to 600-msec) CAD and ETD mass spectra recorded during data-dependent analyses (nHPLC-μESI-MS/MS) of phosphopeptides generated in a tryptic digest of human nuclear proteins.** All peptides were converted to methyl esters and subjected to immobilized metal affinity chromatography before analysis by MS. *A*, CAD spectrum dominated by fragment ions corresponding to the loss of phosphoric acid and either methanol or water. *B*, ETD spectrum containing 13 of 14 possible c- and z-type product ions. Note that the spectrum is devoid of fragment ions corresponding to the loss of phosphoric acid. Copyright 2004 National Academy of Sciences.

To circumvent the difficulties with thermal electron storage in RF fields that would be required, the idea was to use anions with low electron affinities as electron vessels. In 2004, an LTQ in the laboratory was quite famously modified for ETD with a modified nano-ESI source on the front and the addition of a chemical ionization source on back side of the instrument to produce anthracene anions (15, 16, 17, 18). In collaboration with Thermo Electron, the instrument control software was manipulated to allow for the sophisticated movement and co-trapping of peptide cations and anions allowing for this gas phase chemistry to occur. Syka *et al* showed the benefits of ETD fragmentation as compared to CAD when analyzing phosphorylated peptides (16) (Fig. 2). At the end of this seminal ETD publication, several future directions were outlined including the potential for incorporating multiple ion-reactions into individual experiments and applications to larger peptides and intact proteins.

Optimization of ETD on the LTQ instrument continued and follow-up publications also demonstrated the sequencing of intact proteins with this in-house modified instrument and implementation of another ion-ion reaction for proton transfer charge reduction also known as ion-ion proton transfer (IIPT) (19, 20). IIPT reduced the charge of multiply charged ETD fragment ions and therefore simplified the MS/MS spectrum proving valuable for not only intact protein analysis but also large peptide analysis for combinatorial PTM characterization. ETD was commercialized and available on the LTQ from Thermo Electron in 2006. The LTQ was a small benchtop QLT instrument that could be more accessible than existing FTMS and now provided flexibility in fragmentation with both CAD and ETD. Additional insight into the development and applications of ETD will be provided elsewhere in this edition (Story *et al* and White *et al*).

In 2007, Coon, now in his own laboratory at the University of Wisconsin, in collaboration with Thermo Electron, demonstrated ETD implementation on an LTQ-Orbitrap. A negative chemical ionization source was coupled to the C-trap by an octupole at the rear to transport anions to the QLT (21). The first commercial Orbitrap mass spectrometer (LTQ-Orbitrap) was released by Thermo in 2005 and an ETD source based on the one in the Coon laboratory was available as an optional upgrade on the LTQ-Orbitrap XL in 2008. The LTQ-Orbitrap XL also had HCD fragmentation in addition to CAD providing more options for fragmentation on a single high resolution mass spectrometer. Multiple fragmentation methods were possible in a single acquisition including decision-tree automation (22).

In 2006, the front-end ETD source development journey began in the Hunt Laboratory. The goal was to develop a robust and efficient ETD source that could be compatible with any instrument with an atmospheric pressure ionization source. Several approaches were considered and in the end, a glow discharge-based reagent ion source utilizing azulene was developed that was much smaller and brighter than the existing commercial back-end sources (23). A version of this source was implemented on both the LTQ-Orbitrap and LTQ-FT in the Hunt Laboratory. Additionally, through collaboration, a version of the source was installed on a 21-Tesla FT-ICR mass spectrometer at the National High Magnetic Field Laboratory (24).

During the time between the first ETD paper and the front-end ETD source, the Orbitrap instrument lines continued to evolve with updated ion optics and enhanced Orbitraps. In 2013, the first tribrid Orbitrap instrument was released. This marked the commercial introduction of the front-end ETD anion source based on the longstanding collaboration with the Hunt Laboratory. This provided a very robust anion source and improved ETD fragmentation. It also improved utilization of multiple fragmentation methods in a single analysis. It still took until the Eclipse tribrid instrument release in 2019 for a charge reduction ion-ion reaction to be available in the Thermo commercial instrument line with the introduction of proton transfer charge reduction (IIPT) utilizing perfluoroperhydrophenanthrene.

## Top-Down Proteomics

The front-end ETD source opened the door wider for advancements in intact protein analysis. Hunt laboratory efforts and advancements in collaboration with Finnigan/Thermo for intact protein sequencing will be discussed in this edition by Story *et al*. The front-end ETD source improved intact protein analysis by allowing multiple fills of the C-trap with ETD product ions increasing the ion current allowing for faster analysis of intact proteins. IIPT was optimized to simplify ETD spectra and disperse fragment ions over the entire mass providing improved ion coverage across protein lengths (25). Additionally, derivatization techniques were developed to improve ion coverage in both the far C- and N-terminal regions of protein sequences which were often poorly covered. A combination of ETD, IIPT, and derivatization of the amino and acidic groups resulted in a total sequence coverage for apomyoglobin, a 153 amino acid–containing protein, of 94% (25).

At this same time, utilization of parallel ion parking (PIP), first reported by McLuckey et al. (26, 27), was being implemented on the ETD-enabled high resolution mass spectrometers in the laboratory. PIP is a method used to selectively inhibit ion-ion reactions based on m/z ranges. When a precursor has undergone sufficient IIPT reactions to reduce the charge state to a desired m/z range, PIP can be used to slow the rate or additional reactions and therefore parking the ions at a charge state. A collaboration around 2015 between the Hunt laboratory and the laboratory of alum Ben Garcia was published showing the power of ETD, IIPT, and PIP when applied to the complexity of histone protein analysis (28).

In 2019, the implementation of a modified PIP technique on an in-house–modified Orbitrap Elite was published (29). The early utilization of PIP on the QLT-Orbitrap instruments in the laboratory used AC waveforms that were not as effective at selectively suppressing ion-ion reactions for the high charge state product ions of intact proteins. Initial efforts to improve this process included increasing the amplitude of the POP waveform, but this resulted in collision-induced fragmentation of the charge-reduced protein product ions. The updated approach involved kinetic excitation of the product ion m/z range with simultaneous kinetic activation of the reagent ions to slow the overall kinetics of IIPT (29). This improved method was applied to the intact analysis of *E. coli* ribosome, which consists of 54 proteins ranging from 3.5 to 61 kDa in size. In total, 46 proteins were identified from a ProSight PD (Thermo) search, 6 from manual MS/MS interpretation, and the remaining 2 were identified by intact mass as they were not selected for HCD fragmentation.

In 2021, another publication from the lab demonstrated the application of PIP during ETD, known as ETD-PIP, to improve sequence analysis of intact proteins (30). This work was also performed on the in-house–modified Orbitrap Elite. Protein G, a 21 kDa protein, was used for proof-of-concept experiments showing an increase in sequence coverage from 80% with ETD alone to 90% with ETD-PIP. In addition, 90% of complementary ion pairs were observed with ETD-PIP as compared to 39% with standard ETD. The combination of different ion-ion reactions, PIP, and other techniques have advanced intact protein analysis by leaps and bounds over the past roughly 25 years. The Hunt laboratory has contributed to this area through the development of instrumentation, optimization of techniques, and collaboration with other experts in this area. If interested in recent work in this area, refer to the research articles by Hinkle *et al* and Hakansson *et al* included in this edition.

Don’s interests in mass spectrometer technology developments were inspired by his desire to delve into complicated biological questions. He saw gaps in the available technology when it came to studying peptides and proteins in complex biological specimens and worked towards building the technology and tools needed. Don also has an ability to recognize the potential of others’ work and utilize it in new ways. He has always been interested in sharing these advancements and is well known for teaching many mass spectrometrists formally and informally about ETD and how to interpret MS/MS spectra generated from this fragmentation method. For further discussion of Don’s role of teaching countless individuals how to *de novo* sequence peptides from CAD and ETD spectra, refer to the Anderson *et al* in this edition.

## Answering Complex Biological Questions

Just as Don’s work in instrumentation was motivated by a desire to advance biological applications of proteomics, the same can be said for his interest in pre-analytical method development that would improve mass spectrometric analysis of complex mixtures. Don’s goal was not to develop instrumentation and techniques that were specific to one question or one study but to build robust systems that could be applied to diverse biological investigations routinely. Below are a few select examples of Hunt laboratory research focus areas that exemplify the larger themes of Don’s career and demonstrate how his work contributed to the growth of the field of MS-based proteomics.

### Immunopeptidomics

Don saw great potential in the application of MS for the study of immunology, and the extensive contributions of the Hunt laboratory in this domain will be further highlighted elsewhere in this edition (Abelin *et al*). Don was specifically interested in identifying antigen peptides presented on major histocompatibility complexes (MHC) for the purpose of finding potential targets for immunotherapy with a focus on cancer. A long collaborative relationship with Victor Engelhard, also at the University of Virginia, was well established by 2000. The laboratories had been working together to understand immunological questions including identifying MHC peptides as part of the Engelhard laboratory’s focus on studying the recognition of antigens by CD8 T cells. In 1992, this collaboration led to the sequencing of the first MHC peptides and in 1998, the identification of the first phosphorylated MHC peptide from a cancer cell line (31, 32). The identification of a phosphorylated MHC peptide meant that there was potentially a new set of tumor-specific antigen targets as proteins in cancer cells had already been shown to be differently phosphorylated.

Then in 2000, the two labs published a large study in which multiple MHC class alleles were enriched in a sequential manner followed by Fe^3+^ immobilized metal affinity chromatography (IMAC) for phosphorylated peptide enrichment (33, 34). This large study demonstrated how biologically important peptides present at extremely low levels could be isolated and then sequenced by MS. Fe^3+^ IMAC was not a novel technique nor was its application to phosphorylated protein enrichment, however, its use to identify phosphorylated antigen peptides started a long and impactful effort to discover cancer-specific phosphorylated MHC peptides (35, 36).

### Phosphoproteome

Besides the exciting explorations into phosphorylated MHC peptides, the Hunt laboratory was more generally interested in improving the detection of phosphorylated peptides and proteins due to the important role of this modification on protein function and regulation of biological processes. In 2002, Ficarro *et al* published a paper demonstrating the application of an improved Fe^3+^ IMAC procedure for analysis of the *Saccharomyces cerevisiae* phosphoproteome in which 216 phosphopeptides from 171 different proteins were identified and manually confirmed (37). This publication introduced the use of chemistry for the conversion of carboxylic acid side chains to methyl esters prior to Fe^3+^ IMAC to minimize nonspecific binding during the enrichment step. This was not new chemistry, but it was a novel application. Following phosphorylated peptide enrichment, the sample was analyzed *via* LCMS, and an in-house neutral loss software was used to help identify more than 1000 phosphorylated serine- and threonine-containing peptides by the neutral loss of the phosphoric acid group during CAD. The data was also searched using SEQUEST and then spectra were manually validated to confirm phosphorylation sites. The ability to detect phosphorylated peptides present at substoichiometric levels in complex samples provided a pathway for studying the vast impacts of phosphorylation on protein function.

Many other experts in the field were also exploring different ways to improve the detection of phosphorylated peptides and proteins by MS. These techniques included derivatization, enrichment, and often multidimensional MS techniques (38, 39, 40). However, Fe^3+^ IMAC with methyl ester derivatization was shown to virtually eliminate nonspecific binding, minimize sample loss, enriches serine, threonine, and tyrosine phosphorylated peptides, and could be modified for relative quantitation between two samples with the use of deuterated methanol (41). The conversion of carboxylic groups to methyl esters prior to Fe^3+^ IMAC became a widely utilized method in the Hunt laboratory and was applied to study many other biological questions of interest.

By 2004, the technology and methodology existed in the laboratory to perform LTQ-FT analysis for accurate mass precursor measurements and a second experiment on the ETD enabled LTQ for sequence information and site localization on IMAC-enriched phosphorylated MHC peptides (42). By 2009, a single analysis was possible on the LTQ-FT or LTQ-Orbitrap with front-end ETD allowing for a single LCMS analysis to provide a robust data set (43, 44). The optimization of IMAC enrichment in the laboratory combined with high resolution MS and ETD MS/MS enabled discovery of many novel phosphorylated immunopeptides and biologically important phosphorylation sites more generally.

### Epigenetics and the Histone Code

Another major area of research over the decades in the Hunt laboratory was focused on understanding the role of histone proteins in gene activation and gene silencing or more simply epigenetics. Don’s earliest work in this area was in the early 1990’s in collaboration with Juan Ausio at University of Victoria to characterize a histone H1-like protein found in the sperm of *Mytilus californianus* (45, 46). This collaboration continued through the entirety of Don’s career. In the early 2000’s, the Hunt laboratory also collaborated with the Pemberton laboratory at the University of Virginia to study histone nuclear transport by identify proteins that interacted with histones in the cell (47). In the early 2000’s, Don formed a collaborative relationship with David Allis and his laboratory (University of Virginia and later Rockefeller University) (48).

Although the important role of the Hunt laboratory contributions to histone biology will be discussed in greater detail later in this edition (Ueberheide *et al*), some major highlights need to be mentioned. First, the application of high-resolution MS to this field allowed for differentiation between the many highly modified isoforms of histone proteins. Histone proteins, especially H3 and H4, contain an unusually high number of lysines and arginines. If digested by trypsin, many of the peptides would be too small to be compatible with LCMS methodology. Additionally, histone H3 and H4 are highly modified on lysines and arginines and many of these modifications will result in missed cleavages and inconsistent peptide generation. In 2002, a chemical derivatization method utilizing propionic anhydride to convert amines to propionyl amides was introduced by the Hunt laboratory. This method reduced the basicity of the peptides and added a hydrophobic moiety to every free N-termini, unmodified, and mono-methylated lysine (12, 49). This was especially impactful on the profiling of PTMs on the very basic histone H3 and H4 proteins. The derivatization resulted in all lysines being modified and therefore not cleaved by trypsin, so more reproducible peptides were produced.

Additionally, with histone proteins, the goal was to be able to profile the different combinations of PTMs on an individual histone protein and that required larger peptides. If histone H3 was digested with endoproteinase Glu-C, it would generate a 50 amino acid N-terminal piece that carries a vast majority of the PTMs (50). ETD fragmentation was a perfect tool for the large and highly charged histone peptides generated by digestion by endoproteinases like Glu-C. Profiling of the combination of the PTMs on the same histone protein has led to groundbreaking discoveries and understanding of gene regulation. Additionally, ETD made it possible to identify and determine site localization of more labile PTMs such as phosphorylation. Utilization of these techniques went on to provide great insight into the roles of histone proteins in the differently modified forms. These types of analysis only improved with the further advancements of intact protein analysis (28).

### O-GlcNAc, the “Other” Labile Modification

Another large focus during this time frame in the laboratory was efforts into identifying and profiling O-GlcNAc (51). Identification and mapping of O-GlcNAc sites posed an analytical challenge as the modification is usually present at a substoichiometric level, modified peptides co-elute with their unmodified counterparts, presence of the unmodified peptide suppresses ionization of the O-GlcNAcylated peptide, and modification is very labile. Therefore, enrichment was required, but early attempts using antibody-based strategies were not efficient and lectin techniques were not specific. Development chemoenzymatic approaches utilizing biotinylation by Jerry Hart’s laboratory improved the enrichment of this biologically important PTM (52). Use of a photocleavable biotin reagent optimized in collaboration between the Hart and Hunt laboratories increased the recovery of the peptides but also had benefits for MS analysis (53). It enhanced O-GlcNAc site mapping using ETD by adding a charged moiety to the peptide and provided a means to screen for modified peptides using CAD fragmentation as the tag produced two signature fragment ions. Techniques developed through this collaboration resulted in the mapping of O-GlcNAc sites in many biologically important pathways (51, 54). Other colleagues (Udeshi *et al*) will provide an in-depth exploration into the development of methods for the study of this labile and extremely complex PTM and its biological significance.

### Cell Migration

In 2001, The Cell Migration Consortium launched as a large-scale collaborative initiative sponsored by the National Institute of General Medical Science (NIGMS) and it was spearheaded by Rick Horwitz and Tom Parsons also at the University of Virginia. The CMC brought together investigators from over a dozen institutions to work toward a better understanding of how cells move. Cell migration is required for higher organism development but also plays a large role in certain pathologies (55). The goal of this consortium was to bring together scientists with diverse expertise to develop and implement standardized assays, provide access to advanced techniques, and share information. This initiative aligned well with Don’s goals and interests. Additionally, the Hunt laboratory’s success in protein analysis in complex mixtures and advancements in phosphorylated peptide analysis made them a key partner in this collaborative venture.

A major focus of the Hunt laboratory contribution to The Cell Migration Consortium was the identification and or confirmation of known, predicted, and novel phosphorylation sites on proteins in key pathways. During this large-scale collaboration from 2001 to 2013, the instrumentation in Don’s laboratory continuously advanced benefiting the greater efforts of the consortium. Fe^3+^ IMAC and improvements on the procedure expanded the phosphorylation site knowledgebase. Additionally, the Hunt laboratory served as advisors on how to design experiments and prepare the appropriate samples to maximize the potential of MS analysis. Some excellent examples from this large-scale effort include phosphorylation site identification on talin, paxillin, FAK, identification of Ras-interacting proteins, and many other contributions (56, 57, 58, 59, 60, 61, 62, 63, 64, 65). Many of the techniques and procedures honed while part of the CMC were more widely applied through countless collaborations at UVA and beyond leading to insights into complex biological processes.

## Conclusions

**Fig. 3:**
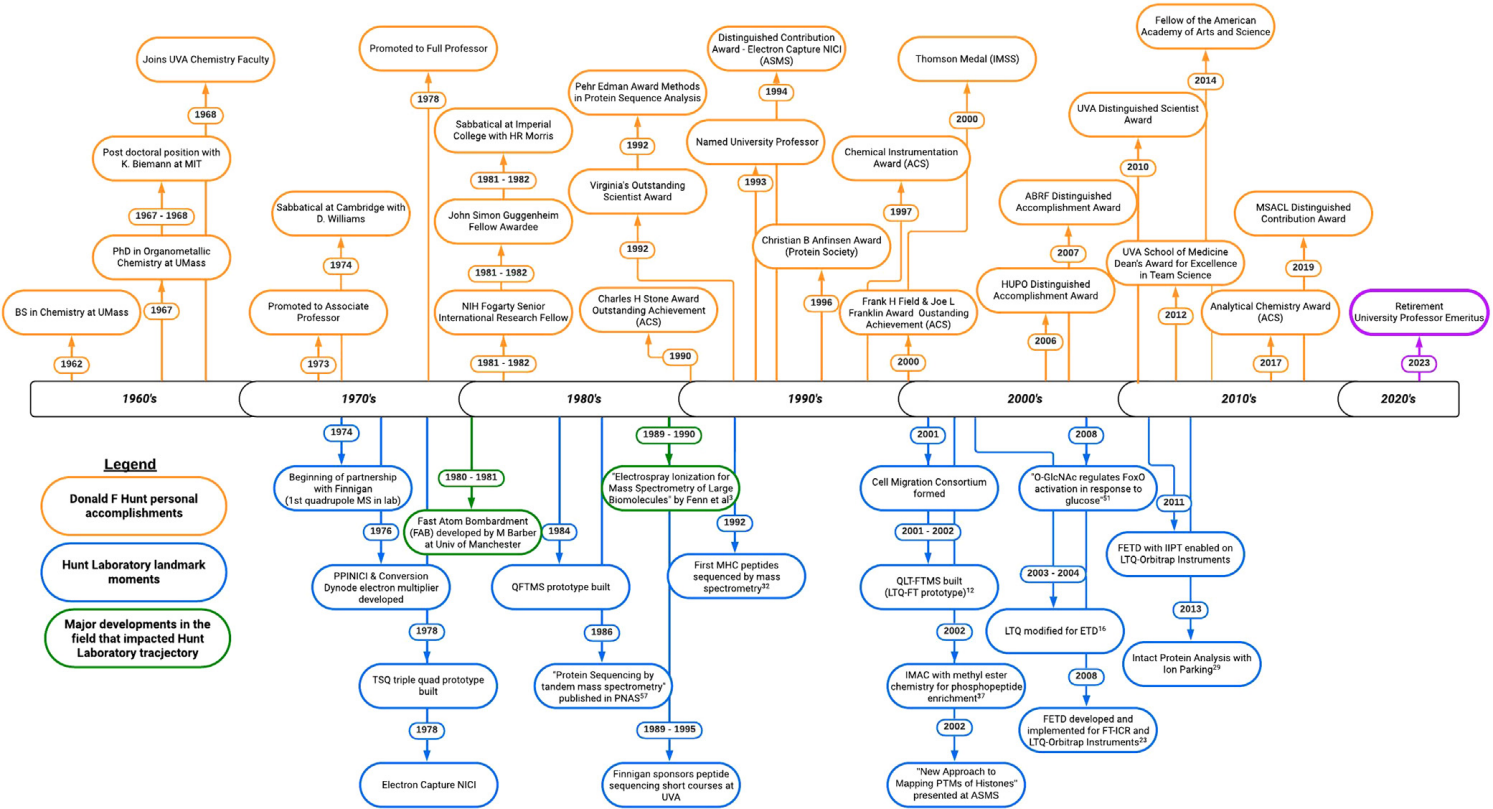
**Timeline of Donald F Hunt and the Hunt laboratory major milesones.** Personal achievements and awards are in *orange*, and significant laboratory moments are in *blue*. Select developments in the field that had a large impact on the trajectory of the laboratory's work are in *green*.

During his career, Don built strong collaborative relationships with colleagues who had different expertise but shared interest in complex biological questions. His goals were to build tools and methods to interrogate many complicated systems. He often saw the untapped full potential of techniques others conceived and found unique applications. Don’s work and the Hunt laboratory contributions over the years is a notable example of the power of collaboration as the developments of technology and chemistry by this group has spread out to the larger scientific community and provided paths for the study of previously inaccessible subjects. The included timeline (Fig 3) highlights many of Don's professional accomplishments and major milestones of the Hunt Laboratory in an effort to visually display his impact.

**Fig. 4:**
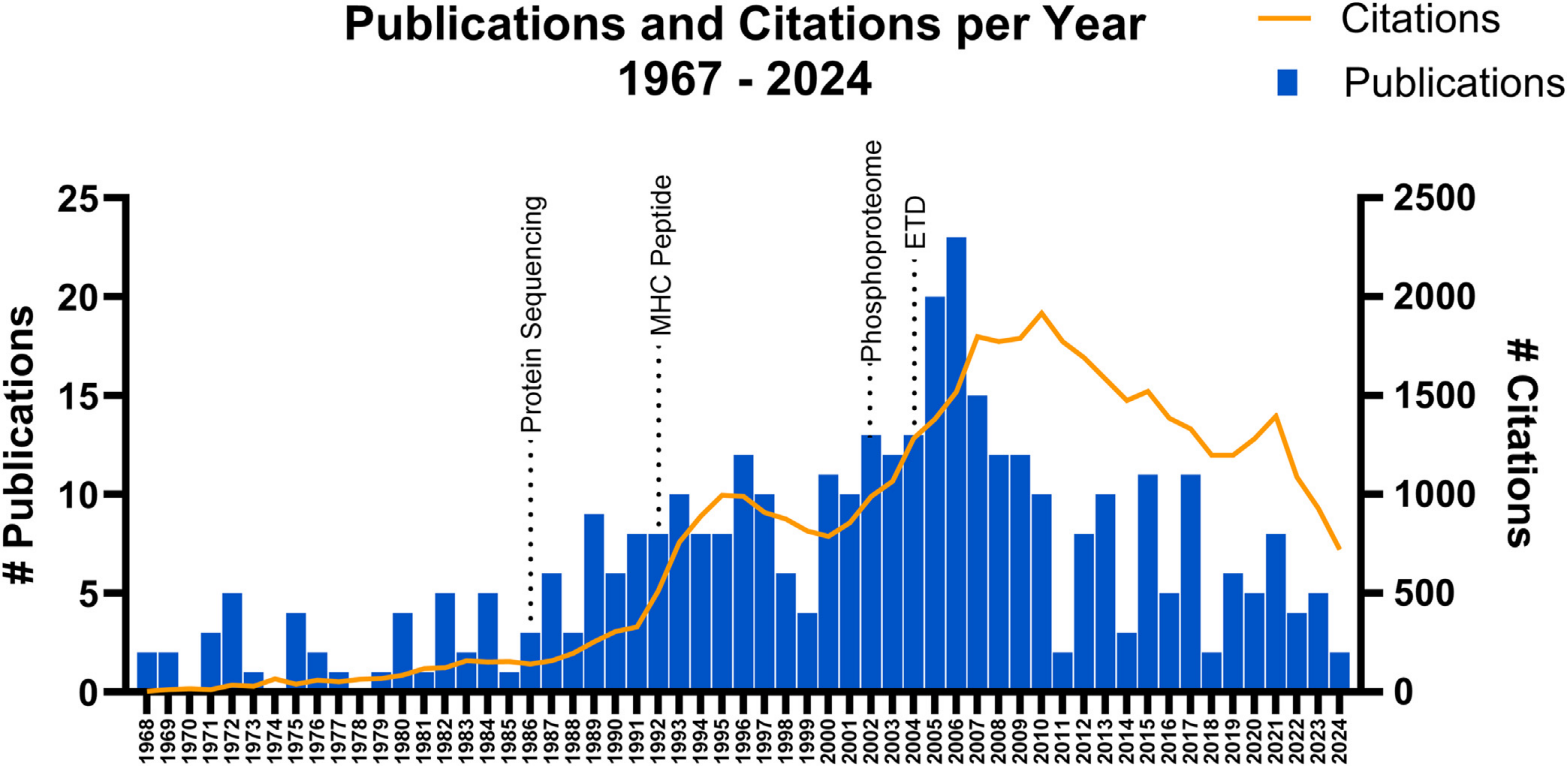
**A summary of Hunt laboratory publications and citations by year from 1968 to 2024 highlighting the four most highly cited publications over 56 years.** The most cited publication with a total of 1956 citations was published in 2004 and introduced electron transfer dissociation as implemented on an LTQ (16). Ficarro *et al* published the paper about phosphoproteome analysis including Fe^3+^ IMAC in 2002 and it has been cited 1406 times (37). The 1986 publication describing the methodology for peptide sequencing using mass spectrometry has been cited 1122 times (66). “Characterization of peptides bound to the class-I MHC molecular HLA-A2.1 by mass spectrometry” which was published in 1992 has been cited 1118 times (32). Certain data included herein are derived from Clarivate (Web of Science). Clarivate 2024. All rights reserved. Data is from Web of Science and was collected on October 11, 2024 using the author search term DF Hunt and filtered to only include articles.

Movement of MS-based proteomics into the clinical sphere is happening as Don had predicted (1). Although it has been slower than many had hoped, Don should be proud to know that he has played a part in that evolution. Thank you for training me and making it possible for me to be one of the people playing a small part in bringing proteomics to patient care diagnostics.

It is hard to encapsulate the impact Don has had on the field, but he has graduated 93 PhD students, 7 MSc students, mentored 27 post-doctoral scientists, and welcomed 8 visiting scientists since he started his laboratory at the University of the Virginia in 1968. To search through the many members of the Hunt Laboratory follow this link to an academic tree (https://academictree.org/chemistry/peopleinfo.php?pid=63541). Additionally, Don has been an author on over 400 publications which have been cited roughly 43,000 times (Fig. 4). The most cited is the 2004 ETD paper followed by the Fe^3+^ IMAC phosphoproteome study published in 2002. A peptide sequencing with MS methodology paper from 1986 and the first MHC peptide sequencing publication round out the four most cited works correctly highlighting some of the most impactful moments in the Hunt Laboratory. These highlights cannot summarize Don’s contributions to the field himself or the impacts of his mentees and collaborators, but the articles included in this edition suggest the influence of his work.

## Conflicts of interest

The authors declare no competing interests.
